# JAK/STAT Signalling in Huntington’s Disease Immune Cells

**DOI:** 10.1371/currents.hd.5791c897b5c3bebeed93b1d1da0c0648

**Published:** 2013-12-13

**Authors:** Ulrike Träger, Anna Magnusson, Nayana Lahiri Swales, Edward Wild, Janet North, Mark Lowdell, Maria Björkqvist

**Affiliations:** Department of Neurodegenerative Disease, Institute of Neurology, University College London, London, UK; Department of Experimental Medical Science, Wallenberg Neuroscience Center, Lund University, Lund, Sweden; University College London, London, UK; Institute of Neurology, University College London, London, UK; Bristol University, Bristol UK; University College London, London, UK; University College London, London, UK; Department of Experimental Medical Science, Wallenberg Neuroscience Center, Lund University, Lund, Sweden

## Abstract

Huntington’s disease (HD) is an inherited neurodegenerative disorder caused by a CAG repeat expansion in the huntingtin (HTT) gene. Both central and peripheral innate immune activation have been described as features of the disease. Isolated human HD monocytes have been shown to produce more cytokines upon LPS stimulation compared to control monocytes. Understanding alterations in the signalling cascades responsible and activated by this increase in pro-inflammatory cytokine production is crucial in understanding the molecular basis of this phenomenon. Here we investigated the signalling cascade most commonly activated by pro-inflammatory cytokines such as IL-6 – the JAK/STAT signalling cascade. Using flow cytometry, we show that one out of three key transcription factors activated by JAK/STAT signalling is altered in primary human HD innate immune cells, suggesting that this pathway may only play a minor, additive role in the immune cell dysfunction in HD.

## Introduction

Huntington's disease (HD) is a fatal, autosomal dominant, progressive neurodegenerative disorder that results from the expansion of a trinucleotide CAG repeat within the *HTT* gene that encodes a protein called huntingtin (HTT) . The disease is characterised by severe neuronal loss, causing a progressive loss of cognitive, psychiatric and motor function 1.

While primary pathology is thought to arise from neuronal dysfunction and death, HTT expression has been found in every tissue studied 2 and many reports demonstrate abnormalities in peripheral tissues 3. HTT is also expressed in immune cells 4 ^,^ 5 and both central and peripheral innate immune cells have been shown to be abnormal in HD patients. Activation of microglia, the macrophages of the brain, has been shown in post-mortem HD brain tissue 6 and by PET imaging of premanifest HD gene carriers 7 .We have previously reported peripheral immune system dysfunction in the form of changes in levels of innate immune proteins such as complement factors and cytokines in HD patient plasma 8. Elevated cytokine 4 and chemokine 9 levels found in HD patients correlate with disease progression and can be detected as early as 16 years before disease onset. Furthermore, stimulation of both murine and human primary *ex vivo* monocytes with LPS leads to abnormally high production of IL-6, suggesting a hyper-reactive phenotype in HD myeloid cells 4 . Mutant HTT interacting with the key kinase of the NFκB pathway – IKK – has been shown as one cause of increased cytokine production in primary human HD immune cells, by leading to increased activation of the NFκB signalling cascade upon stimulation with LPS 10.

The JAK/STAT (janus kinase/ signal transducer and activator of transcription) signalling pathway is a key element in the communication between immune cells and is mainly activated through cytokine receptors 11 . The binding of cytokines to their receptors leads to the activation and cross-phosphorylation of JAK tyrosine kinases, which are bound to the intracellular domain of the cytokine receptor. This activation leads to phosphorylation of tyrosine residues in the intracellular domain of the receptor. STAT signalling molecules then bind these phosphotyrosine residues with their SH2 domain and are subsequently phosphorylated by JAKs, allowing the STATs to dimerise via a phosphotyrosine-SH2 interaction and translocate into the nucleus to act as transcription factors and induce transcription of cytokine genes 12 ^,^ 13. There are different STAT family members (STAT1-6), which are activated by different receptors and different JAKs. This leads to the expression of different genes, depending on which STAT has been activated. For instance, STAT1 is activated by IFNγ, whilst STAT3 is activated by IL-6 and IL-10 signalling, and STAT5 is activated by GM-CSF or IL-2 14. Both STAT3 and, to a lesser extend, STAT1 are activated in monocytes stimulated by IL-6, while STAT5 has been linked to monocyte production of IL-6 14 ^,^ 15.

In this study, we aimed to examine whether the elevated plasma cytokine levels found in HD patients 4 can lead to chronic activation of the JAK/STAT pathway downstream of the cytokine receptors in HD monocytes. Therefore, using a flow cytometry approach, the levels of activated, phosphorylated (p)STAT signalling molecules were compared between primary human *ex vivo* HD and control monocytes. IL-6 was the key cytokine found to be abnormal in HD patient plasma 4, therefore we choose to study STAT1, STAT3 and STAT5 given their link to IL-6. We investigated both the effect of increased levels of IL-6 on JAK/STAT signalling in monocytes, and if increased IL-6 production by monocytes could be linked to JAK/STAT signalling.

## Methods

**Collection and classification of human samples**

All human experiments were performed in accordance with the Declaration of Helsinki and approved by the University College London (UCL)/UCL Hospitals Joint Research Ethics Committee (LREC 03/N008). All subjects provided informed written consent. Blood samples were obtained from control subjects and genetically-diagnosed HD patients, recruited from the Huntington's disease clinic at the National Hospital for Neurology and Neurosurgery, London.

Premanifest HD mutation carriers were identified according to the absence of diagnostic motor abnormalities on the UHSRS 16, whilst patients with motor abnormalities were classed as having early or moderate-stage disease using the total functional capacity scale (13-7, early; 6-3, moderate) 17. Subjects with inflammatory or infective conditions were excluded.

**Phosphoflow analysis of STAT phosphorylation**

Peripheral blood mononuclear cells (PBMCs) were isolated from blood samples using density centrifugation, by layering the samples on top of Lympholyte cell separation media (Cedarlane). After spinning for 30 min at 400x g the layer of PBMCs was carefully transferred into a fresh, sterile 50 ml centrifuge tube and washed with HBSS (spin for 5 min at 300x g). In order to remove platelets, cells were washed with 25 ml HBSS (spin at 200x g for 10 min). Cell pellets were resuspended in 5 ml HBSS and counted using a Neubauer counting chamber. 1x10^6^cells were distributed in six FACS tube (BD Biosciences) each per subject and each tube was filled up to 1 ml with HBSS before resting the cells for 30 min at 37°C followed by trituration and resting for additional 10 min at 37°C. After centrifugation at 300x g for 5 min, the pellets were vortexed before 5 μl of anti- human CD64 antibody (BD Pharmingen), diluted 1 in 3 in HBSS, were added and incubated for 15 min in the dark at room temperature. Samples were washed with 1 ml HBSS (spin 300x g for 5 min) before stimulation. For each subject, three tubes remained unstimulated with the addition of 1 ml fresh HBSS and one tube was stimulated with either 20 ng/ml IFNγ, IL-6 or GM-CSF in 1 ml HBSS. Samples were incubated for 20 min at 37°C before adding 1 ml 37°C pre-heated BD Phosflow Cytofix buffer and incubating at 37°C for an additional 10 min. After centrifugation at 300xg for 5 min, the supernatants were poured off and 1 ml -20°C cold BD Phosflow Perm Buffer III was added and the samples were incubated for 30 min in the dark on ice. The samples were then washed twice with 1 ml BD Phosphoflow stain buffer (centrifuged at 300x g for 5 min). Finally, samples were stained intracellularly with Alexa Fluor 647 anti- human pSTAT antibodies (10 μl for pSTAT1, 5 μl for pSTAT3 and pSTAT5; BD Biosciences) for 30 min in the dark at room temperature. After washing with 2 ml BD Phosphoflow stain buffer (centrifuged at 300x g for 5 min) samples were analysed on a FACScalibur with CellQuest software Pro. Data analysis was performed using FlowJo7.6.5.

Experiments were run over different days, and data for baseline and stimulated pSTAT was normalised to the mean pSTAT of the controls run on the same day (at least two controls were run on each day), to avoid batch effects. To analyse the level of pSTAT activation per subject, pSTAT levels after stimulation were divided by pSTAT levels at baseline, and no normalisation to control took place.

## Results

To assess the level of activated, and therefore phosphorylated, STAT (pSTAT) signalling molecules in HD compared to control cells, blood was collected from a large cohort of control and premanifest HD gene carriers, early and moderate HD patients (demographics shown in Table 1).

**Table 1: HTT CAG repeats length, age and gender ratio for subjects participating in the study.** 

	n	Age	CAG	% female
Control	22	44.2 +/- 16.1	-	45.5
Premanifest HD	18	49.6 +/- 8.3	41.1 +/- 2.5	66.7
Early HD	20	45.9 +/- 12.9	45.8 +/- 5.6	45.0
Moderate HD	11	57.1 +/- 10.1	44.7 +/- 3.4	45.5

PBMCs were isolated by density centrifugation and stained for the monocyte marker CD64. Cells were then either left unstimulated or treated with specific JAK/STAT activators such as IFNγ, IL-6 or GM-CSF to induce STAT1, 3 and 5 phosphorylation, respectively. After staining the cells with intracellular antibodies for pSTAT1, 3 or 5, levels of the proteins were assessed using flow cytometry. For analysis, monocytes were identified by positive CD64 staining, before pSTAT levels were quantified (gating shown in Figure 1).

**Gating for STAT phosphoflow analysis.**
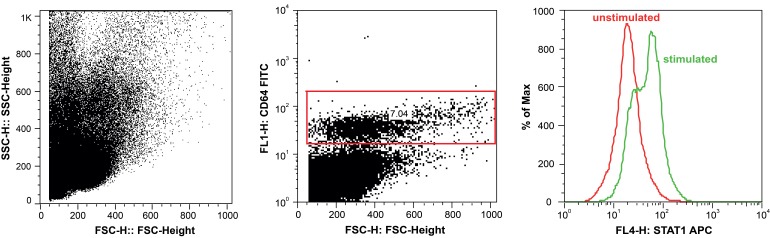
PBMCs were isolated using density centrifugation, stimulated with, for example (as shown here) IFNγ to induce STAT1 phosphorylation. For flow cytometry analysis, monocytes were identified within the PBMC population by gating on CD64 positive cells (middle panel), before pSTAT levels were blotted as histogram (right panel). Overlay of unstimulated and IFNγ -stimulated cells showed a shift in pSTAT expression levels in monocytes. pSTAT levels were quantified as geometric mean. The same gating was used to measure pSTAT3 and 5 levels stimulated by IL-6 or GM-CSF, respectively.

Comparing pSTAT levels measured in untreated* ex vivo *control and HD patient cells showed no difference in the levels of phosphorylated STAT1, 3 or 5 (Figure 2), suggesting no effect of chronic exposure to elevated plasma cytokines on JAK/STAT signalling in HD patient monocytes. Similarly no significant differences were observed between control and HD cells, after stimulating the JAK/STAT pathway with IFNγ, IL-6 or GM-CSF for STAT1, 3 and 5, respectively (Figure 2).

**STAT signalling appears normal in HD subjects’ monocytes.**
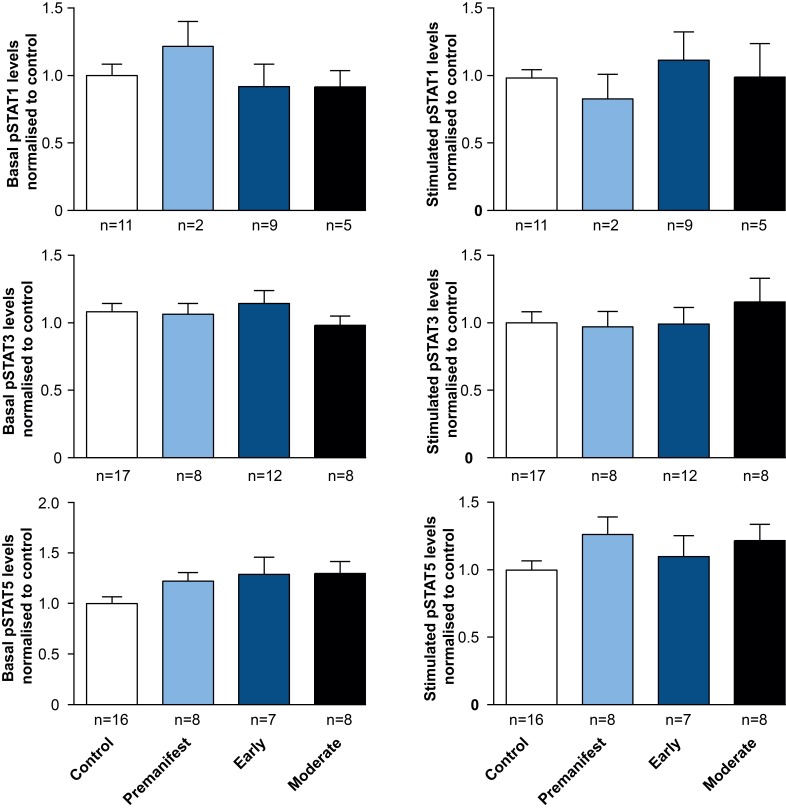
PBMCs were isolated using gradient density centrifugation, stimulated with either IFNγ (for STAT1 activation), IL-6 (for STAT3 activation) or GM-CSF (for STAT5 activation) and stained for CD64 and pSTAT1, 3 and 5. Flow cytometry was used to assess the amount of pSTAT molecules in untreated and treated CD64^+^ monocytes. Data is shown as mean pSTAT levels normalised to control levels +/- SEM. One-way ANOVA was used as statistical measure and no statically significant difference was found.

In order to increase the subject number for the analysis, we combined the data from HD subjects of all the different disease stages. By doing this, we could show significantly, although only slightly increased levels of pSTAT5 in baseline HD monocytes compared to controls (Figure 3). However, after stimulation of monocytes there were no significant differences detectable.

**pSTAT5 levels are increased in HD patient monocytes at baseline.**
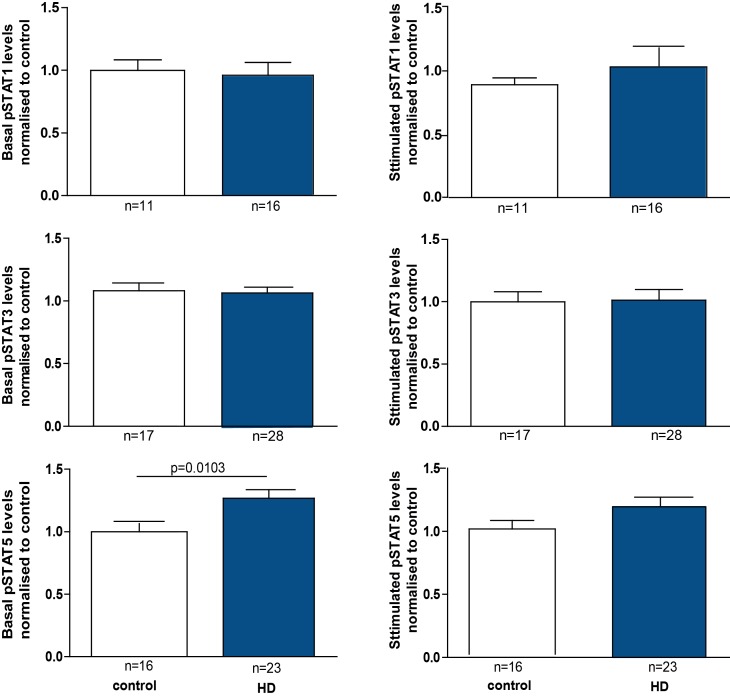
PBMCs were isolated using gradient density centrifugation, stimulated with either IFNγ (for STAT1 activation), IL-6 (for STAT3 activation) or GM-CSF (for STAT5 activation) and stained for CD64 and pSTAT1, 3 or 5. Flow cytometry was used to assess the amount of pSTAT molecules in untreated and treated CD64+ monocytes. Data is shown as mean pSTAT levels normalised to control levels +/- SEM. Two-way student t test was used for statistical analysis.

In addition to comparing total levels of activated pSTAT molecules at baseline and in stimulated monocytes, the fold change of activation was calculated by dividing the level of pSTAT after stimulation by the pre-stimulation level. While robust activation of the JAK/STAT pathway was observed upon activation in both control and HD patient cells, comparing the fold change activation between HD and control cells did not show a difference between groups (Figure 4).

**Activation of STAT signalling appears normal in HD patient monocytes.**
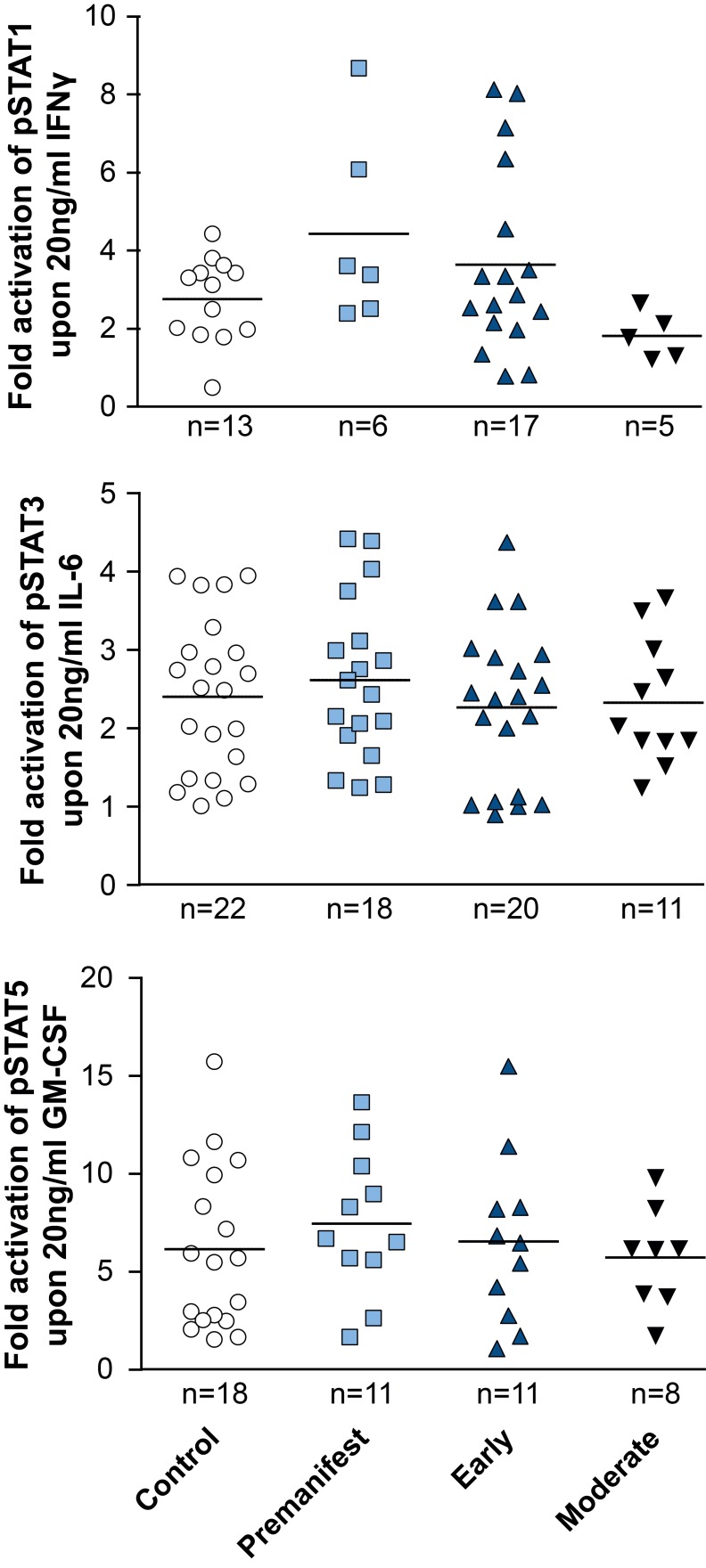
PBMCs were isolated using density centrifugation, stimulated with IFNγ (for STAT1 activation), IL-6 (for STAT3 activation) or GM-CSF (for STAT5 activation) and stained for CD64 and STAT1, 3 and 5. Flow cytometry was used to assess the amount of pSTAT molecules before calculating the fold-activation by dividing post-treatment pSTAT levels by pre-treatment levels. Data shown as mean fold change +/-SEM. One-way ANOVA was used as statistical measure and no statistically significant difference was found.

## Discussion

The most commonly activated signalling pathway downstream of cytokine receptors is the JAK/STAT pathway, coordinating cytokine-mediated gene expression and repression 12. Plasma pro-inflammatory cytokine and chemokine levels are elevated in HD patient plasma, even during the premanifest stage of the disease 4 ^,^ 9. In this study we investigated the activation of the JAK/STAT pathway by measuring STAT phosphorylation in *ex vivo* monocytes isolated from HD patients and controls. We used a flow cytometry based technique to analyse the phosphorylation state of STAT molecules, “phosphoflow”, that has been widely used in different fields to access activation of different pathways such as MAPK and STAT signalling on a single cell basis 18 ^,^ 19 ^,^ 20.

While both pSTAT1 and pSTAT3 levels were unchanged in HD gene carriers/monocytes from all disease stages compared with control cells at baseline, pSTAT5 levels were significantly elevated at baseline in HD monocytes, though not in any individual subgroup of HD mutation carriers. Targeted activation of JAK/STAT signalling molecules using specific STAT1, 3 and 5 activators (IFNγ, IL-6 and GM-CSF, respectively) demonstrated the same level of pathway activation in control and HD monocytes, indicating normal function of the signalling cascade in primary human HD immune cells upon stimulation.

The increase in baseline pSTAT5 levels in HD monocytes, may affect other signalling cascades that are known to interact with the JAK/STAT signalling pathway. Using a murine meroblastic leukaemia cell line expressing constitutively active STAT5A, Kawashima et al. demonstrated that IL-3 and GM-CSF can activate STAT5, which in turn enhances the DNA-binding activity of NFκB leading to IL-6 production 21. This known cross-link between STAT5 and NFκB suggests an additive effect of the two pathways. Interestingly, NFκB signalling pathway has been shown to be abnormal in HD 10 ^,^ 22. An additive effect of the small changes in baseline STAT5 activity presented in this study combined with that of NFκB could contribute to the increase in IL-6 production observed in HD cells. Though we find no evidence for it in this limited study, we therefore cannot exclude the possibility that STAT signalling further enhances the increased NFκB activation found in HD 22 ^,^ 23.
